# Alterations in the red blood cell membrane proteome in alzheimer's subjects reflect disease-related changes and provide insight into altered cell morphology

**DOI:** 10.1186/1477-5956-8-11

**Published:** 2010-03-03

**Authors:** Joy G Mohanty, Hem D Shukla, Jefferey D Williamson, Lenore J Launer, Satya Saxena, Joseph M Rifkind

**Affiliations:** 1 National Institute on Aging, Baltimore, MD, USA; 2 Wake Forest University, Winston-Salem, NC, USA; 3 National Institute on Aging, Bethesda, MD, USA

## Abstract

**Background:**

Our earlier studies have shown that red blood cell (RBC) morphology in Alzheimer's disease (AD) subjects was altered (> 15% of the RBCs were elongated as compared to 5.9% in normal controls (p < 0.0001)). These results suggested alterations in the RBC membrane architecture in AD subjects, possibly due to RBC-β-amyloid interactions and/or changes in the expression of membrane proteins. We hypothesized that the observed changes could be due to changes in the level of the protein components of the cytoskeleton and those linked to the RBC membrane. To examine this, we performed a proteomic analysis of RBC membrane proteins of AD subjects, and their age-matched controls using one pool of samples from each group, following their separation by SDS-PAGE, in-gel Tryptic digestion, LC-MS-MS of peptides generated, and a label-free approach of semi-quantitative analysis of their relative MS spectral intensities.

**Results:**

The data suggest, (1) RBC shape/morphology changes in AD subjects are possibly attributed primarily to the changes (elevation or decrease) in the level of a series of membrane/cytoskeleton proteins involved in regulating the stability and elasticity of the RBC membrane, and (2) changes (elevation or decrease) in the level of a second series of proteins in the RBC membrane proteome reflect similar changes reported earlier by various investigators in AD or animal model of AD. Of particular interest, elevation of oxidative stress response proteins such as heat shock 90 kDa protein 1 alpha in AD subjects has been confirmed by western blot analysis in the RBC membrane proteome.

**Conclusions:**

The results suggest that this study provides a potential link between the alterations in RBC membrane proteome in AD subjects and AD pathology.

## Background

Alzheimer's disease (AD) is a progressive neurodegenerative disorder characterized by abnormal extracellular deposition of β-amyloid (Aβ) peptide and neuronal loss. Recently, we have analyzed red blood cell (RBC) morphology in blood from subjects with AD and reported [1] that > 15% of the RBCs are elongated as compared to 5.9% in normal controls (p < 0.0001). This observation suggests possible alterations in the RBC membrane architecture in AD subjects. These changes are likely to originate from modifications caused by Aβ interactions with RBCs [2,3] and/or changes in the expression level of certain RBC proteins.

It is well established [4] that the cytoplasmic surface of the RBC plasma membrane contains a two-dimensional meshwork of proteins referred to as the spectrin membrane cytoskeleton. Attached to integral membrane proteins, this cytoskeleton provides elasticity, flexibility and stability to the RBC, as the cells continuously flow through the circulatory system. Passage through the narrow blood vessels and micro capillaries involve large changes in shear stress and significant deformation of the RBC. Because of the ease in obtaining RBCs and the lack of internal RBC organelles, the plasma membrane of these cells has been extensively studied. The identity, function, and topology of many RBC membrane proteins have been determined [4-6]. A number of proteins associated with the RBC cytoskeleton have been shown to alter the shape of RBCs. Therefore, we hypothesized that the observed increase in RBCs with a non-biconcave shape from AD subjects [1], could be traced back to changes in the level of the protein components of the cytoskeleton and the proteins linking the cytoskeleton to the membrane.

A recent review published by Goodman *et. al*. [7], compiled the proteomic data on RBC proteins gathered over the last five years by several laboratories. These studies identified 751 proteins within the human erythrocyte, including about 340 membrane proteins reported by Pasini *et. al*. in 2006 [8]. Many of the identified proteins were shown to play a role in regulating the shape and stability of the RBC. Therefore, we performed a proteomic analysis of RBC membrane proteins of AD subjects, and their age-matched controls, to see if we can explain the observed RBC shape changes in AD subjects [1], and to determine whether RBC membrane proteomics reflect changes in the level of proteins linked to AD pathology.

## Results and Discussion

### LC-MS-MS data from RBC membrane proteins

**Figure 1:**
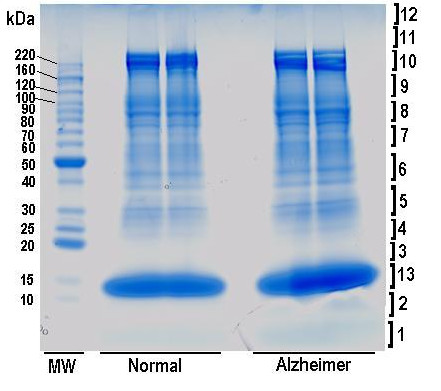
**SDS-PAGE of RBC membrane proteins**. Sample of coomassie blue stained SDS-PAGE gel containing RBC membrane proteins from AD (Alzheimer) subjects and age matched controls (Normal) from Run 1. The numbers on right side of the gel indicate slice numbers or the area where gel was cut. Numbers of left side of the gel indicates molecular size (MW) in kilo Daltons (kDa).

As described in the Methods section below, membrane proteins were prepared from two RBC pools (AD and normal controls) consisting of 5 subjects in each pool. Pooling samples shown by statistical analysis [9] to decrease noise and, thereby, increase the reliability of the determination, has been used in a proteomic analysis of bronchoalveolar lavage proteins [10]. The membrane proteins in control and Alzheimer pooled samples were isolated using three different approaches: (1) membrane preparation in pH 7.4 buffer followed by solubilization with 2%SDS (Run 1); (2) membrane preparation in pH 8.0 buffer (pH 8 minimizes haemoglobin binding to the membrane) followed by solubilization with 2%SDS (Run 2); and (3) membrane preparation in pH 7.4 buffer followed by solubilization with SIGMA Protein Extraction Reagent Type 4 (normally used for 2D electrophoresis, Run 3). Each of these preparations of membrane proteins was resolved through SDS-PAGE. After staining and de-staining of the gels, sample lanes were sliced as shown in Figure 1 (Run 1), and subjected to in-gel trypsin digestion of proteins prior to elution of peptides for analysis by LC-MS-MS. Thus, there were three sets of data obtained from the analysis of proteins prepared by three different approaches (Runs 1-3). By incorporating three different methods of RBC membrane protein isolation in this study, conclusions from RBC proteome analysis were believed to be independent of the methods used.

### Calculation of Spectral Intensity ratios from LC-MS-MS data

We detected a total of 785 proteins in normal and AD samples using LC-MS-MS data of three runs and their analysis with Agilent's Spectrum Mill software as described in the methods. The autovalidation feature of this software is unique and has been successfully used by several investigators [11-13]. Some proteins were found to be present in one, two or all three runs of both AD and control samples, while some were found only in AD or control samples. There were also significant differences in the levels of certain proteins for AD and control samples. Several criteria were used to determine which proteins displayed a significant increase or decrease in their level in samples from AD subjects in comparison to samples from normal control subjects. (1) Protein entries with only one peptide match - considered insignificant - were removed. This step removed 170 proteins, leaving 615 proteins for further consideration. (2) To make the data more reliable, proteins with a unique score less than 25 were deleted. This step reduced the number of proteins to 548. (3) Proteins detected only in one run - considered insignificant - were eliminated. This step reduced the list of proteins to 224. (4) Among these 224 proteins, three accession numbers had no specific protein name or little available information - and so, were not considered in the present study. (5) Among the rest of the 221 proteins, there were two proteins observed only in AD subjects, while 26 proteins were observed solely in normal subjects. The proteins exclusively present or absent in samples from AD subjects represent potential biomarkers. However, the identification of a biomarker would require validation for the protein assignment and a demonstration that the changes were present in a number of individual subjects. Such an analysis was beyond the scope of this study and will be considered in a future publication.

**Figure 2:**
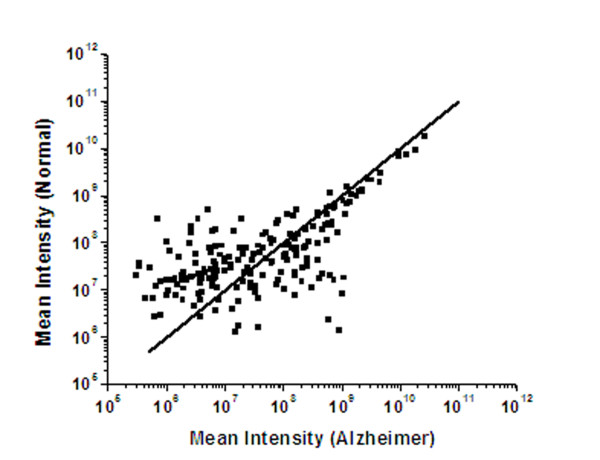
**Comparison of Mass Spectral intensities in normal and AD samples**. Comparison of the mean Mass-spectral intensities of proteins (observed in two or three runs) in the RBC-membrane proteome in normal and AD subjects. The line was generated for equivalent intensities in both groups of subjects. Points to the right of the line indicate proteins with elevated intensities in the AD subjects and points to the left of the line indicate proteins with elevated intensities in normal subjects.

In this study, we focused our attention on the 193 proteins detected in both AD and control samples. Although, care was taken to minimize contamination of platelets and nucleated cells during RBC isolation, minor contamination may still be possible, which might contribute to these 193 proteins. The mean intensities of these proteins are compared for AD subjects and controls using data from equivalent number of runs from each group (Figure 2 scattered points). The line shown in Figure 2 was generated for equivalent intensities in both groups of subjects. Points to the right of the line indicate proteins with elevated intensities in the AD subjects and points to the left of the line indicate proteins with elevated intensities in normal subjects. Figure 2 indicates that there are a number of proteins with appreciable differences in intensities when both groups are compared. We focused our analysis on those proteins. To evaluate significant changes between AD and normal subjects, the total spectral intensities for each of the 193 proteins were added separately and the ratios of the corresponding total intensities (AD/normal) were calculated. Thus, for a particular protein, a ratio value greater or lesser than 1 would indicate an increase or decrease respectively in its level in the AD subjects. However, to make our data more reliable, we considered a ratio of 1.5 or more (1.5-fold or more increase) for a protein to be significantly increased in AD subjects. Similarly, a ratio of 0.67 or lower (1.5-fold or more decrease) for a protein was considered significant to conclude that the level of that protein decreased in AD subjects. Thus proteins with a ratio of greater than 0.67 and less than 1.5 were eliminated from further consideration.

These above criteria reduced the number of proteins being considered to 160. These proteins along with the change in their respective level (AD/normal), their IPI accession numbers, and their linked gene names are presented in a separate table as Supplementary material (Additional file 1). Most of the proteins listed in this table have been previously reported to be present in the RBC proteome [7]. Proteins shown in bold were reported to be present in the RBC membrane proteome by Pasini *et. al*. [8], and proteins with asterisks have been reported to be present in the whole RBC proteome by Goodman *et. al*. [7]. To analyze further, we have focused on two groups of proteins, one that is involved with RBC cell morphology and the other, which has been reported by other investigators in AD as well as in animal models of the disease.

### Level of Membrane/Cytoskeleton proteins linked to RBC morphology are altered in AD subjects

There is evidence for the formation of RBCs with altered morphology in AD subjects [1,14,15], which reflect perturbations impairing the required deformability of the RBC. This deformability is accomplished by a cytoskeleton on the cytoplasmic side of the RBC membrane that consists of spectrin tetramers (2α and 2β chains). The cytoskeleton is linked to the membrane by a network of proteins that participate in both horizontal and vertical interactions [16]. The spectrin hetero-dimer is anchored to the plasma membrane via two vertical attachment sites. The first attachment site is assembled by the band 3-ankyrin complex that binds to a site near the carboxyl terminus of β-spectrin, providing a mechanism, by which the "head" region of the spectrin hetero-dimer binds to the cytoplasmic surface of the plasma membrane [16-18]. This linkage is strengthened by the protein band 4.2. The second vertical bridge is located at the "tail" region of the spectrin hetero-dimer and is composed of several proteins collectively known as the "junctional complex" [19,20]. This region of spectrin is associated with actin protofilaments, which are bound to adducin and tropomyosin and linked to glycophorin C via band 4.1 and dematin (band 4.9). Tropomodulin-1 is a well defined actin-capping protein that interacts with tropomyosin at the pointed end of actin filaments [21]. This junctional complex has been shown to play a critical role in the maintenance of erythrocyte elasticity, shape and membrane stability [16,18,20]. Changes in the level of one or more of these proteins may contribute to decreased deformability of RBC, leading to the decrease in blood flow through the microcirculation, impaired oxygen delivery and consequently to AD pathology [1,22].

Our proteomic study of the RBC membrane in AD subjects makes it possible to explain these morphology/shape changes. In Table 1, we have listed the proteins involved in the cytoskeleton network that are altered in AD subjects relative to the normal controls. Our results suggest that the level of band 4.2 protein increased by 70% in AD subjects. This increase may alter the proper balance between band 4.2 and the link of the membrane to the spectrin head. With respect to the junctional complex involving the spectrin tail, there are significant changes in the level of a number of proteins including tropomodulin, tropomyosin, adducin, glycophorin, dematin, and the F-actin capping protein. In addition, we have also observed changes in the level of ezrin which contains the N-terminal band 4.1 sequence involved in binding to glycophorin. Interestingly, all of the above stated proteins were implicated in changes in RBC stability. However, the decrease in adducin and dematin is of particular interest because of a study by Chen et. al. [23], in which it has been shown that the reduction in the level of either dematin or β-adducin exerted modest effects on RBC shape and stability, with a more pronounced effect when both proteins are reduced/eliminated. These changes taken together suggest altered morphology with decreased deformability and stability of the RBCs in AD subjects.

**Table 1:** Alteration in the level of RBC membrane/cytoskeletal proteins related to cell morphology

Accession No.	Fold Change (AD/Normal)	Protein Name
**IPI00028120**	**1.71**	**Erythrocyte membrane protein band 4.2**
**IPI00220741**	**1.83**	**Spectrin alpha chain, erythrocyte**
IPI00216704	2.07	Splice isoform 2 of P11277 Spectrin beta chain, erythrocyte*
**IPI00002375**	**2.88**	**Tropomodulin 1**
**IPI00218319**	**2.15**	**Splice isoform 2 of P06753 Tropomyosin alpha 3 chain (tropomyosin 3 isoform 2)**
IPI00220158	0.43	Splice isoform 3 of P35611 Alpha adducin (Erythrocyte adducin subunit alpha)*
**IPI00026299**	**4.03**	**Splice isoform of P04921 Glycophorin C**
IPI00216311	5.11	Villin 2 (cytovillin 2;, Ezrin) (has high degree of similarity within its N-terminal domain to the erythrocyte cytoskeletal protein, band 4.1, Gould et al., EMBO Journal vol.8 no.13 pp.4133-4142, 1989)*
**IPI00005969**	**7.84**	**F-actin capping protein alpha-1 subunit**
IPI00292290	0.3	Splice isoform Long of Q08495 Dematin (Erythrocyte membrane protein band 4.9)*

N.B. Proteins shown in bold letters have been reported by Pasini et. al. [8] to be present in RBC membrane and those with asterisks have been reported by Goodman et. al. [7] to be present in whole RBC proteome.

### Level of other Membrane/Cytoskeleton proteins linked to Alzheimer's disease are also altered in RBC of AD subjects

There is extensive literature describing changes in protein levels associated with AD. Most of these studies are focused on brain tissue in both human and animal models. It is, possible to observe similar changes in RBC membrane if the activation or inhibition of protein synthesis observed in AD brains affect protein synthesis in other tissues including RBCs. Alternatively, the observed changes in RBC membrane from AD subjects may reflect proteins that are taken up by RBCs when they come in contact with these tissues [24-26]. For both of these reasons, changes in RBC membrane proteins can be used to monitor changes in AD pathology. Therefore, we searched the literature to find if any of the proteins in our RBC membrane proteome (Additional file 1) have any link to AD. We looked for correlations with the reported change in human AD subjects or in an animal model of AD. Proteins, showing similar changes in both our study as well as in existing AD literature are listed in Table 2.

**Table 2:** Alteration in the level of RBC membrane/cytoskeletal proteins similar to those alterations in AD or its animal model

Accession No.	Fold Change (AD/Normal)	Protein Name	Citations showing Similar fold changes
IPI00008274	8.13	Adenylyl cyclase-associated protein	[27]
IPI00010314	1.78	Aminolevulinate, delta-, dehydratase	[30]
**IPI00024689**	**2.61**	**Aquaporin-CHIP (Aquaporin-1, AQP-1, AQP1)**	[36]
IPI00027462	3.38	Calgranulin B (S100A9)	[37]
IPI00233820	2.61	Catalase*	[54,55]
**IPI00027438**	**1.89**	**Flotillin-1**	[38]
**IPI00328602**	**123.42**	**heat shock 90 kDa protein 1, alpha**	[33,56]
IPI00217966	634.23	L-lactate dehydrogenase A*	[39]
**IPI00298860**	**2.88**	**Lactotransferrin precursor (Lactoferrin) [Contains: Lactoferroxin A**	[40]
**IPI00218342**	**31.93**	**Methylenetetrahydrofolate dehydrogenase 1 (MTHFD1)**	[41]
**IPI00022434**	**2.08**	**Serum albumin precursor**	[43]
IPI00180818	0.18	Aldolase A *	[44]
IPI00026119	0.19	Ubiquitin-activating enzyme E1*	[45]

N.B. Proteins shown in bold letters have been reported by Pasini et. al. [8] to be present in RBC membrane and those with asterisks have been reported by Goodman et. al. [7] to be present in whole RBC proteome.

**Figure 3:**
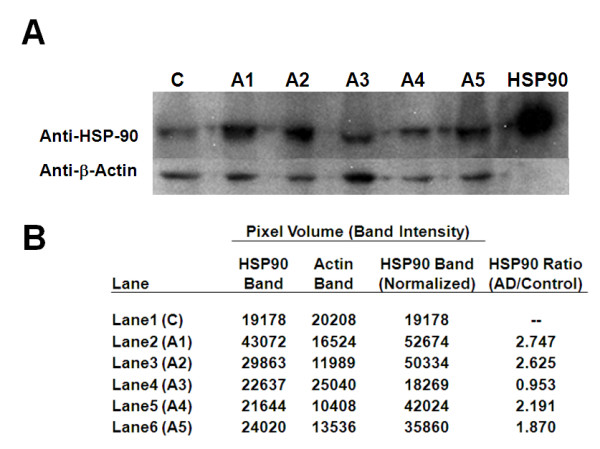
**Validation of increase in HSP90 protein in RBC membrane proteome of AD subjects**. (A) Western blot analysis of HSP90 and beta-actin protein in RBC membrane samples from pooled control (C) and five AD subjects (A1-A5). (B) After normalization of actin band intensities in AD samples (A1-A5) with respect to actin band in control sample, ratios of HSP90 band intensities in AD vs. control show elevation of HSP90 in four AD subjects (A1, A2, A4 and A5). The positive control band of HSP90 is shown next to A5 (heavy band).

Adenylyl cyclase-associated protein, a transport/cytoskeleton protein, was found to be 2.2 fold up-regulated in presenilin knockout mice having neurodegeneration [27]. Oxidative stress has been implicated in AD and has been reported to be elevated in presenilin knockout mice independent of brain Aβ deposition [28,29]. Aluminium-induced oxidative stress in rat brain was shown to increase the level of aminolevulinic acid dehydratase (ALAD) in blood [30]. Hypoxia, known to cause oxidative stress, was elevated in clinical AD [31], causing an increase in stress response proteins such as heat shock 90 kDa protein 1 alpha (HSP90) [32,33]. For HSP90 we confirmed our MS data (Table 2) using western blot analysis of RBC membrane proteins with a monoclonal HSP90 antibody, showing an elevation of HSP90 in four out of five AD subjects (Figure 3). An increase in the oxidative stress in AD is expected to result in an elevation [34,35] of catalase in AD subjects as we noticed (Table 2). Aquaporin 1 (AQP1) expression in the cerebral cortex was shown to have been increased at early stages of Alzheimer disease [36]. Calgranulin B (S100A9), an inflammation-associated protein was reported to be expressed in AD but not in normal human brains [37]. According to Girardot *et. al*. [38], confocal microscopy evidence suggests that flotillin-1 was accumulated most often in tangle-bearing neurons in AD subjects. A report showed a significant increase in specific activity of lactate dehydrogenase A in frontal and temporal cortex of AD brains [39]. Lactotransferrin expression was reported to be up-regulated in both neurons and glia in affected AD tissue [40]. It was reported that methylenetetrahydrofolate dehydrogenase 1 (MTHFD1) genotype had a tendency of increased frequency in AD subjects [41]. According to Hye *et al*. [42], serum albumin precursor was elevated in plasma from AD subjects and a similar elevation was observed in brain of mouse model AD [43]. In contrast, we have also observed two proteins - aldolase A and Ubiquitin-activating enzyme E1 - whose levels were decreased in RBC proteome of AD subjects similar to that reported in AD hippocampus proteome [44], and AD brain samples respectively [45].

## Conclusions

The present membrane proteomics data suggest two aspects of how changes in the level of proteins in RBC membrane can contribute to our understanding of AD. (1) The shape/morphology changes observed earlier in the RBC of AD subjects are most likely attributed primarily to the changes in the level of a series of membrane/cytoskeleton proteins that are involved in regulating the shape, stability and elasticity of the RBC membrane. In turn, these changes can alter RBC deformability and impair oxygen transport in AD subjects. (2) The changes in the level of a second series of proteins in our RBC membrane proteome reflect similar changes reported earlier by other investigators in AD subjects or animal models of AD. For some of these proteins, such as HSP90 and L-lactate dehydrogenase A, the changes are very dramatic and may be useful for AD diagnosis. We validated the increase of HSP90 in the RBC membrane proteome from AD subjects by using western blot analysis (Figure 3A &3B). Thus, this study provides a potential link between the RBC membrane proteome changes in AD subjects and AD pathology. The potential significance of this study is indicated by the fact that we have been able to detect differences even with subjects in the early stage of AD with only very mild dementia.

## Methods

### Sample Collection

This investigation of RBC membrane proteome changes in AD subjects was a pilot study of 10 subjects (5 AD subjects and 5 controls) as part of a National Institute on Aging (NIA) approved study "Immune and Other Bio-Markers of Alzheimer's Disease" (NIA protocol # 2005-095), which was included in the Wake Forest University School of Medicine Ginkgo Evaluation of Memory Study [46] (GEMS), as reported earlier [1]. Briefly, each AD case was identified based on their scores on a preliminary battery of 3 tests (3MSE, Modified Mini-Mental State Examination (scale 1 to 100) [47], CDR, Clinical Dementia Rating [48] and ADAS, Alzheimer's Disease Assessment Scale [49]) followed by a full neuropsychological battery (NPB), a standardized examination by a neurologist and a brain magnetic resonance imaging (MRI) scan. All these data were then used by a panel with expertise in dementia diagnosis to confirm dementia incidence and classify according to dementia subtype. This procedure made it possible to diagnose subjects in the early stage of AD. Thus, all AD subjects were at an early stage of AD with very mild dementia (a CDR of 0.5 and 3MSE score as low as 70). Control cases were matched for age and cardiovascular disease history with 3MSE score of 90 or above. None of the subjects reported any incidence of disease that required hospitalization including cardiovascular disease such as angina, Congestive heart failure (CHF), stroke/Transient ischemic attack (TIA) or Peripheral vascular disease (PVD) during 6 months prior to sample collection. Characteristics of all participants in our RBC proteome study as regards their age, gender, medications used are listed in Table 3.

**Table 3:** Personal and medication data of AD (A1-A5) and control (C1-C5) subjects used in this study:

Subject	Gender	Age	Medications taken
A1	Female	81	ESTRGN

A2	Female	84	ACED, NTCA, THRY, HTNMED

A3	Male	89	ASA

A4	Male	99	ADPI, ASA, HCTZ

A5	Male	85	ALZH

C1	Female	83	ASA, BETA, LOOP, DIUR, HTNMED

C2	Female	91	ASA, COX2, THRY

C3	Male	87	ASA, DLTIR, NSAID, CCBIR, CCB, HTNMED

C4	Male	87	HCTZ, PPI, DIUR, HTNMED

C5	Male	85	None

N.B. Medication abbreviations are ACED (Ace inhibitors with diuretics), ADPI (Inhibitors of ADP-induced platelet aggregation), ALZH (Acetylcholine esterase inhibitors for AD), ASA (Aspirin), BETA (Beta-blockers without diuretics), CCB (Any Calcium-channel blocker = CCIR OR CCBSR OR CCBT), CCBIR (Immediate-release CCBS = NIFIR OR DIHIR OR VERIR OR DLTIR), CCBT (T-type Calcium-channel blocker), COX2 (COX-2 inhibitors (NSAID Agents); separate from NSAID variable), DIHIR (Immediate-release Dihydropyridines other than Nifedipine), DIUR (Any diuretic), DLTIR (Immediate-release Diltiazem), ESTRGN (Estrogens, excluding vaginal creams), HCTZ (Thiazide diuretics without K-sparing agents), HTNMED (Any anti-hypertensive medication), LOOP (Loop diuretics), NIFIR (Immediate-release Nifedipine), NSAID (Non-steroidal anti-inflammatory agents, excluding Aspirin), NTCA (Non-tricyclic antidepressants other than MAO Inhibitors), PPI (Proton pump inhibitors), THRY (Thyroid agents), VERIR (Immediate-release Verapamil),

Blood samples from five AD subjects and five age-matched controls were collected in heparinised (green cap) tubes at Roena Kulynych Center, Wake Forest University School of Medicine (Winston-Salem, North Carolina). Blood was centrifuged at low speed (~1125 × g) to remove plasma. The pelleted cells were washed three times (each time a portion of top layer of RBC removed to minimize contamination of nucleated cells) with phosphate buffered saline (PBS) containing 100 μM disodium EDTA. Washed red blood cells (pellets) were shipped frozen on dry ice to our NIA laboratory at Baltimore for further processing. Samples upon receipt were stored at -150°C.

### Isolation of membrane proteins

Membrane proteins were isolated from RBC pellets shipped overnight in dry ice and stored frozen at -150°C. Frozen RBC pellets were thawed in the presence of 1 mM (final conc.) phenyl methyl sulfonyl fluoride (PMSF) a serine protease inhibitor by adding a stock solution of 100 mM PMSF in ethanol to the cell pellet. PMSF is well documented as an irreversible serine protease inhibitor in the pH range of 4 to 8.5 [50,51] and has been used in proteomic studies [52]. After thawing, 100 μl aliquots of the cell pellet from each of the 5 control samples and from each of the five Alzheimer's samples were pooled separately resulting in a final volume of 500 μl for each category. In order to take into account potential variability in membrane protein solubilization, three different conditions (Runs 1-3) were used to prepare membranes and solubilize them. Cells in the pool of RBC pellets were lysed by diluting them by the addition of 9 ml of 10 mM Phosphate buffer, pH 7.4 (Runs 1 and 3) or 9 ml of 5 mM Phosphate buffer, pH 8.0 (condition used to prepare white ghosts [6] (Run 2)). In each buffer, inhibition of proteolysis was also maintained by the addition of PMSF (1 mM final conc.). Hemolysates were centrifuged either at ~39,000 × g (Runs 1 and 3) or at ~9000 × g [6] (Run 2) for 30 min. Membrane pellets were resuspended in 10 mM Phosphate buffer, pH 7.4 (Runs 1 and 3) or 5 mM Phosphate buffer, pH 8.0 (Run 2) and washed by centrifuging at ~39,000 × g (Runs 1 and 3) or at ~20,000 × g [6] (Run 2) until the supernatant was clear. Membrane pellets were solubilized by vortexing with 500 μl of either of 10 mM Tris.HCl, pH 7.4 containing 2% SDS (Runs 1 and 2) or SIGMA Protein Extraction Reagent Type 4(Run 3) containing urea, thiourea, Tris, and detergent C7BzO. Insoluble debris was removed by spinning at ~39,000 × g (Runs 1 and 3) or at ~14,000 × g [6] (Run 2) for 30 min. Protein assay in the clear supernatants from runs 1 and 2 were performed using BCA reagent from Pierce, while protein assay in the clear supernatant from run 3 was performed using BIORAD Bradford reagent, since urea and thiourea interfered with the BCA assay.

### SDS-PAGE Analysis

Samples with ~50 μg (Runs 1 and 2, two lanes each) or 100 μg (Run 3, one lane each) per lane of RBC membrane proteins from control and Alzheimer's subjects were subjected to SDS-Polyacrylamide gel electrophoresis (SDS-PAGE) under reducing conditions on commercial small gradient (NOVEX, 4-20%) gels (Runs 1 and 2) or on regular size gradient gel (Jules Inc., 4-20%) (Run 3). Following electrophoresis, gels were stained with coomassie blue. Then each gel was carefully laid on a glass plate and horizontal slices were cut with new razor blades for each sample. Each slice was cut into ~1 mm cubes and the pieces were transferred to the corresponding labelled-microfuge tube.

### Tryptic digestion

Gel pieces were destained with 25% acetonitrile in 25 mM ammonium bicarbonate (three times), and were dehydrated with 400 μl of 100% acetonitrile followed by vacuum drying. Proteins in the gel pieces were then subjected to reductive alkylation by adding 10 mM DTT in 5 mM ammonium bicarbonate, incubating the tubes at 55°C for 45 min, subsequently aspirating the liquid, and incubating the gel pieces with 150 μl of 55 mM iodoacetamide in the same buffer in the dark. Residual DTT and iodoacetamide were washed off the gel pieces with 5 mM ammonium bicarbonate (four times) and dehydrated for 10 min by the addition of 400 μl of 100% acetonitrile. Proteins in gel pieces were then digested overnight with Trypsin Gold, (Mass Spectrometry Grade) from Promega (trypsin: protein was about 1:20) at 37°C to obtain the corresponding peptides. The peptides were then eluted from gel pieces with 50% Acetonitrile, 45% HPLC grade water, and 0.5% formic acid (two times) and once with 100% acetonitrile. The eluted fractions were pooled, dried in a SpeedVac (Savant ThermoFisher), and dissolved in 10 μl of sample buffer (5% acetonitrile, 0.1% formic acid, 97.9% HPLC grade water).

### LC-MS-MS

Samples (10 μl) were injected by an auto-injector (CTC-Autosampler, CTC Analytics) with a flow rate of 500 nl/min into a LTQ linear ion trap mass spectrometer (ThermoElectron) for LC-MS-MS analysis as described by Johanssen *et. al*. [53]. During LC, a linear gradient from 95% mobile phase A (2% acetonitrile, 0.1% formic acid, 97.9% HPLC grade water) to 90% mobile phase B (2% HPLC grade water, 0.1% formic acid, 97.9% acetonitrile) was used for 110 min followed by 95% mobile phase A for 10 min. The peptides were detected in positive ion mode in the mass spectrometer using a data-dependent method in which the seven most abundant ions detected in an initial survey scan were selected for MS-MS analysis. Each scan cycle consisted of one full scan mass spectrum (m/z 400-1800) collected in profile mode followed by six MS-MS events in centroid mode.

### Peptide Database Search

Raw MS-MS data were analyzed by Spectrum Mill proteomics software (Rev A03.02.060, Agilent Technologies, Santa Clara, CA) after extracting them under default conditions. The extracted files were searched against the human non-redundant International Protein Index (IPI) human sequence database http://www.ebi.ac.uk using trypsin as the protease allowing for a maximum of three missed cleavages, including fixed modification of carbamidomethylation and variable modifications of oxidized methionine and N-terminal glutamine conversion to pyroglutamic acid in the search. Only proteins with at least two validated peptides and a total score 25 or more were considered valid for reporting. To compare identified proteins between AD and normal groups, the number of spectra and the summed ion intensity of peptides for each protein (total ion intensity) were used as indicators of protein amounts. Because these were semi quantitative metrics, we added total intensities for each protein in all three runs for AD and normal subjects separately - then divided them to calculate ratio AD/normal determining if the protein level was elevated (greater than or equal to 1.5) or reduced (less than or equal to 0.67) in AD subjects. Cellular locations of the proteins were determined by using ProteinCenter program version 2.01 (PROXEON, Denmark).

### Western blot analysis

Western blot analysis of HSP90 along with cell standard beta-actin protein was performed on RBC membrane proteins isolated from pooled samples of control subjects and individual samples of five AD subjects prepared as described above. Proteins (50 μg per lane) were separated by SDS-PAGE on NOVEX 4-12% Tris-glycine gels, blotted to PVDF membranes, and probed for HSP90 and beta-actin by using a mouse monoclonal anti-HSP90 (BD Biosciences) and a rabbit polyclonal anti-actin (Santa Cruz Biotechnology) antibody (both at 1:1000 dilution) and WesternBreeze Chemiluminescent Kits (anti-mouse and anti-rabbit) from Invitrogen respectively. Band intensities of HSP-90 and actin bands in the western blot were quantitated upon scanning the blot and measuring the band pixel volume by ImageQuant TL software (GE Healthcare).

## Competing interests

The authors declare that they have no competing interests.

## Authors' contributions

JGM conceived the idea of proteomics study, prepared RBC membrane protein samples, did SDS-PAGE, performed major portion of the sample analysis and drafted the manuscript. HDS performed the gel slicing, in-situ trypsin digestion, LC/MS of peptides and helped in the analysis of the proteomics data. JDW provided the Alzheimer and control blood samples and was involved in setting up the study to compare Alzheimer and control blood samples. LJL participated in the plans to compare Alzheimer and control blood samples. SS oversaw the proteomic analysis of the samples. JMR conceived the study and participated in its design. All authors read and approved the final manuscript.

## Supplementary Material

Additional file 1 List of 160 proteins in RBC proteome with their levels altered (Fold change) in AD subjects in comparison to their matched controls (AD/Normal) along with their IPI accession numbers, and linked gene names. Click here for file
