# Supplementary material for: Alterations in the red blood cell membrane proteome in alzheimer's subjects reflect disease-related changes and provide insight into altered cell morphology
Source: Proteome Sci. 2010 Mar 3;8:11. doi: 10.1186/1477-5956-8-11 (PMC2848146; doi:10.1186/1477-5956-8-11)
Supplement: Additional file 1 — List of 160 proteins in RBC proteome with their levels altered (Fold change) in AD subjects in comparison to their matched controls (AD/Normal) along with their IPI accession numbers, and linked gene names. [file 1477-5956-8-11-S1.PDF]

Ser #	Accession Number	Fold Change (AD/Normal)	Protein Name	Gene
1	**IPI00000816**	**0.09**	**14-3-3 protein epsilon (tyrosine 3-monooxygenase/tryptophan 5-monooxygenase activation protein, epsilon polypeptide)**	YWHAE
2	IPI00219525	0.24	6-Phosphogluconate dehydrogenase, decarboxylating*	
3	IPI00027509	9.06	92 kDa Type IV Collagenase precursor (matrix metallopeptidase 9)	MMP9
4	IPI00008274	8.13	Adenylyl cyclase-associated protein	CAP1
5	**IPI00031131**	**0.24**	**Adipocyte plasma membrane-associated protein (chromosome 20 open reading frame 3) (human RBC acid phosphatase 99% homology IPI00000115)**	C20orf3
6	IPI00007188	0.38	ADP,ATP carrier protein, fibroblast isoform (similar to ADP/ATP translocase 2, ANT 2, ADP,ATP carrier protein 2, Solute carrier family 25 member 5)	SLC25A5 (LOC442525)
7	IPI00215917	5.91	ADP-ribosylation factor 3*	ARF3
8	IPI00180818	0.18	Aldolase A*	ALDOA
9	IPI00305457	0.45	Alpha-1-antitrypsin precursor (Alpha-1 protease inhibitor) (Alpha-1-antiproteinase)	SERPINA1
10	IPI00009253	5.33	Alpha-soluble NSF attachment protein (SNAP-alpha) (N-ethylmaleimide-sensitive factor attachment protein alpha)*	NAPA
11	IPI00010314	1.78	Aminolevulinate, delta-, dehydratase (delta-aminolevulinic acid dehydratase)	ALAD
12	**IPI00024689**	**2.61**	**Aquaporin-CHIP (aquaporin 1 (Colton blood group))**	AQP-1
13	**IPI00291560**	**0.08**	**Arginase type I erythroid variant**	ARG-1
14	IPI00016638	2.95	ATP synthase alpha chain, mitochondrial precursor	ATP5A1
15	**IPI00014555**	**2.43**	**ATP-binding cassette, sub-family B, member 6, mitochondrial precursor**	ABCB6
16	**IPI00021290**	**0.09**	**ATP-citrate synthase (EC 2.3.3.8) (ATP citrate lyase isoform 1)**	ACLY
17	IPI00218019	0.01	Basigin long isoform	BSG
18	IPI00025084	0.11	Calcium-dependent protease, small subunit (calpain, small subunit 1)	CAPNS1
19	IPI00027462	3.38	Calgranulin B (S100 calcium binding protein A9)	S100A9
20	**IPI00020984**	**0.01**	**Calnexin precursor**	CANX
21	IPI00011285	0.16	Calpain 1, large [catalytic] subunit*	CAPN1
22	**IPI00021831**	**0.09**	**cAMP-dependent protein kinase type I-alpha regulatory chain**	PRKAR1A
23	IPI00008388	0.19	CARBONYL reductase*	DCXR
24	IPI00233820	2.61	Catalase*	CAT
25	**IPI00028064**	**0.3**	**Cathepsin G precursor**	CTSG
26	IPI00024067	0.63	Clathrin heavy chain 1*	CLTC
27	IPI00012011	0.31	Cofilin, non-muscle isoform	CFL1
28	IPI00171844	0.06	COP9 subunit 4 (COP9 signalosome complex subunit 4, Signalosome subunit 4, JAB1-containing signalosome subunit 4)*	COPS4
29	IPI00218646	0.39	Cytochrome b-245, beta polypeptide (chronic granulomatous disease)	CYBB
30	IPI00031618	0.04	DNA-damage inducible protein 2*	DDI2
31	IPI00103204	0.29	DnaJ homolog subfamily A member 4 (Hsp40)	DNAJA4
32	IPI00301271	0.01	Dolichyl-diphosphooligosaccharide--protein glycosyltransferase 63 kDa subunit precursor (ribophorin II precursor)	RPN2
33	IPI00025427	1.92	Eosinophil cationic protein precursor (RNAse 3)	RNASE3
34	**IPI00010341**	**0.22**	**Eosinophil granule major basic protein precursor (Proteoglycan 2, natural killer cell activator)**	PRG2
35	**IPI00186072**	**0.23**	**Equilibrative nucleoside transporter 1 (solute carrier family 29 (nucleoside transporters), member 1)**	SLC29A1
36	**IPI00028120**	**1.71**	**Erythrocyte membrane protein band 4.2**	EPB42
37	**IPI00044556**	**0.03**	**Erythroid membrane-associated protein**	ERMAP
38	IPI00376005	0.08	Eukaryotic initiation factor 5A isoform I variant A*	EIF5A
39	IPI00025447	3.06	Eukaryotic translation elongation factor 1-alpha 1*	EEF1A1

40	IPI00232533	0.09	Eukaryotic translation initiation factor 1A, X-chromosomal (X-linked eukaryotic translation initiation factor 1A)	EIF1AP1 (EIF1AX)
41	IPI00219678	2.78	Eukaryotic translation initiation factor 2, subunit 1 alpha, 35kDa*	EIF2S1
42	IPI00297982	0.21	Eukaryotic translation initiation factor 2, subunit 3 gamma, 52kDa	EIF2S3
43	**IPI00220349**	**2.26**	**Eukaryotic translation initiation factor 2C 2**	EIF2C2
44	IPI00298961	0.02	Exportin 1	XPO1
45	**IPI00005969**	**7.84**	**F-actin capping protein alpha-1 subunit**	CAPZA1
46	IPI00294567	0.06	F-box only protein 7*	FBXO7
47	IPI00375676	5.74	Ferritin light polypeptide variant (Hypothetical protein DKFZp686L19147)	FTL
48	**IPI00219910**	**3.96**	**Flavin reductase ((NADPH-dependent diaphorase, NADPH-flavin reductase, Biliverdin reductase B)**	BLVRB
49	**IPI00027438**	**1.89**	**Flotillin-1**	FLOT1
50	IPI00023729	2.19	Fructosamine-3-kinase	FN3K
51	**IPI00219018**	**0.57**	**Glyceraldehyde-3-phosphate dehydrogenase**	GAPDH
52	IPI00004524	0.23	Grancalcin (EF-hand calcium binding protein)	GCA
53	**IPI00288947**	**0.64**	**Guanine nucleotide binding protein (G protein), q polypeptide**	GNAQ
54	**IPI00026268**	**0.51**	**Guanine nucleotide-binding protein G(I)/G(S)/G(T) beta subunit 1**	GNB1
55	**IPI00328602**	**123.42**	**Heat shock 90kDa protein 1, alpha**	HSPCA
56	IPI00384051	0.13	Human full-length cDNA clone CS0DJ015YJ12 of T cells (Jurkat cell line) of Homo sapiens	
57	IPI00016255	0.18	Hypothetical protein LOC79887 (Hypothetical protein FLJ22662)	FLJ22662
58	IPI00218493	0.07	Hypoxanthine phosphoribosyltransferase 1	HPRT1
59	**IPI00385058**	**0.67**	**Ig kappa chain C region (immunoglobulin kappa constant region)**	IGKC
60	IPI00289634	0.22	Importin 9*	IPO9
61	IPI00155940	0.02	Importin alpha 7 subunit (karyopherin alpha 6)	KPNA6
62	IPI00001639	0.08	Importin beta-1 subunit (karyopherin beta 1)*	KPNB1
63	IPI00217987	0.55	Integrin alpha-M precursor	ITGAM
64	IPI00291792	0.37	Integrin beta-2 precursor*	ITGB2
65	IPI00100160	0.32	Isoform 1 of cullin-associated NEDDB-dissociated protein 1 (TIP120 protein, cullin-associated and neddylation-dissociated 1)	CAND1
66	IPI00183503	0.15	Isoform 1 of ubiquitin thioesterase OTU1 (Otubain-1, OTU domain-containing ubiquitin aldehyde-binding protein 1)	OTUB1
67	IPI00334649	261.65	Junction plakoglobin	JUP
68	**IPI00220459**	**5.34**	**Kell blood group glycoprotein (Kell blood group, metallo-endopeptidase)**	KEL
69	IPI00383406	2.49	Keratin 19	KRT19
70	IPI00009865	2.35	Keratin, type I cytoskeletal 10	KRT10
71	IPI00019359	1.92	Keratin, type I cytoskeletal 9	KRT9
72	IPI00217966	634.23	L-lactate dehydrogenase A*	LDHA
73	IPI00219217	0.2	L-lactate dehydrogenase B*	LDHB
74	**IPI00298860**	**2.88**	**Lactotransferrin precursor (Lactoferrin, Lactoferroxin A), growth-inhibiting protein 12**	LTF
75	IPI00217975	0.26	Lamin B1	LMNB1
76	**IPI00219220**	**0.1**	**Lectin, galactoside-binding, soluble, 3 (galectin 3, laminin-binding protein)**	LGALS3
77	IPI00170959	0.11	Leucine-rich repeat-containing protein 57 (Hypothetical protein FLJ37572)	LRRC57
78	IPI00027444	6.73	Leukocyte elastase inhibitor (LEI), Monocyte/neutrophil elastase inhibitor, Serpin B1, serine (or cysteine) proteinase inhibitor, clade B (ovalbumin), member 1	SERPINB1
79	IPI00027769	0.27	Leukocyte elastase precursor (elastase 2, neutrophil pre-protein	ELA2
80	**IPI00291006**	**0.03**	**Malate dehydrogenase, mitochondrial precursor**	MDH2
81	IPI00386401	23.55	Methyl transferase-like protein 7A (Hypothetical protein FLJ13631)	METTL7A
82	**IPI00218342**	**31.93**	**Methylenetetrahydrofolate dehydrogenase 1 (C-1-tetrahydrofolate synthase, cytoplasmic (C1-THF synthase))**	MTHFD1

83	IPI00017596	0.17	Microtubule-associated protein RP/EB family member 1 (APC-binding protein EB1)	MAPRE1
84	IPI00027409	2.35	Myeloblastin precursor (Leukocyte proteinase 3)	PRTN3
85	IPI00013163	2.65	Myeloid cell nuclear differentiation antigen	MNDA
86	IPI00019502	0.31	Myosin heavy chain, nonmuscle type A (Non-muscle myosin heavy chain IIa), Cellular myosin heavy chain, type A	MYH9
87	IPI00374410	0.02	NADH-cytochrome b5 reductase (cytochrome b5 reductase isoform s)	CYB5R3
88	IPI00021827	5.38	Neutrophil defensin 3 precursor (HNP-3) (HP-3) (HP3) (Defensin, alpha 3), defensin, alpha 3 pre-protein	DEFA3
89	IPI00299547	1.72	Neutrophil gelatinase-associated lipocalin precursor (lipocalin 2)*	LCN2
90	IPI00010080	0.05	Oxidative-stress responsive 1*	OXSR1
91	IPI00021085	12.03	Peptidoglycan recognition protein precursor	PGLYRP1
92	IPI00220301	0.25	Peroxiredoxin 6	PRDX6
**93**	**IPI00005181**	**0.47**	**Phospholipid scramblase 1**	PLSCR1
94	IPI00301975	2.96	Placental ribonuclease inhibitor*	RNH1
95	IPI00183626	0.39	Polypyrimidine tract binding protein (polypyrimidine tract-binding protein 1 isoform a)	PTBP1
96	IPI00246058	0.01	Programmed cell death 6-interacting protein (PDCD6IP protein)	PDCD6IP
97	IPI00299000	0.58	Proliferation-associated protein 2G4	PA2G4
98	IPI00219622	0.43	Proteasome alpha 2 subunit*	PSMA2
99	IPI00009949	0.36	Proteasome inhibitor PI31 subunit*	PSMF1
100	IPI00028004	0.09	Proteasome subunit beta type 3*	PSMB3
**101**	**IPI00025252**	**0.36**	**Protein disulfide isomerase A3 precursor, (Disulfide isomerase ER-60, ERp60, 58 kDa microsomal protein, 58 kDa glucose-regulated protein).**	PDIA3
102	IPI00017672	1.83	Purine nucleoside phosphorylase (PNP), Inosine phosphorylase*	NP
103	IPI00305442	20.3	Putative glialblastoma cell differentiation-related protein	GBDR1
104	IPI00031461	0.29	Rab GDP dissociation inhibitor beta (Rab GDI beta), Guanosine diphosphate dissociation inhibitor 2, GDP dissociation inhibitor 2 isoform 1*	GDI2
105	IPI00020436	10.12	Ras-related protein Rab-11B (GTP-binding protein YPT3), RAB11B, member RAS oncogene family*	RAB11B
106	IPI00014577	1.98	Ras-related protein Rab-18, RAB18, member RAS oncogene family*	RAB18
107	IPI00008964	2.38	Ras-related protein Rab-1B, RAB1B, member RAS oncogene family*	RAB1B
**108**	**IPI00007755**	**2.03**	**Ras-related protein Rab-21, RAB21, member RAS oncogene family**	RAB21
**109**	**IPI00016339**	**0.07**	**Ras-related protein Rab-5C, RAB5C, member RAS oncogene family isoform a**	RAB5C
**110**	**IPI00018364**	**2.18**	**Ras-related protein Rap-2b**	RAP2B
**111**	**IPI00028946**	**0.23**	**Reticulon protein 3, reticulon 3 isoform a**	RTN3
112	IPI00020567	0.01	Rho-GTPase-activating protein 1*	ARHGAP1
113	IPI00335085	0.06	Ring finger protein 123	RNF123
114	IPI00298547	3.07	RNA-binding protein regulatory subunit	
115	IPI00305719	0.03	Selenium binding protein 1*	SELENBP1
**116**	**IPI00022434**	**2.08**	**Serum albumin precursor**	ALB
117	IPI00292993	0.48	Similar to bactericidal/permeability-increasing protein	LOC100134379
118	IPI00260020	26.51	Similar to Chain A, Crystal Structure Of The Radixin Ferm Domain Complexed With Inositol-(1,4,5)-Triphosphate*	RDX
119	IPI00328182	7.53	Similar to KIAA0573 protein	KIAA0573
120	IPI00374165	1.57	Similar to pote protein	LOC650770
121	IPI00376109	0.67	Similar to prolyl 4-hydroxylase, beta subunit	LOC728900
**122**	**IPI00220194**	**1.53**	**Solute carrier family 2, facilitated glucose transporter, member 1**	SLC2A1

123	**IPI00216057**	**5.99**	**Sorbitol dehydrogenase (L-iditol 2-dehydrogenase)**	SORD
124	**IPI00220741**	**1.83**	**Spectrin alpha chain, erythrocyte**	SPTA1
125	IPI00028160	0.39	Splice isoform 1 of P08397 Porphobilinogen deaminase (hydroxymethylbilane synthase isoform 1)	HMBS
126	**IPI00003865**	**7.35**	**Splice isoform 1 of P11142 Heat shock cognate 71 kDa protein ((Heat shock 70 kDa protein 8)**	HSPA8
127	IPI00020042	0.14	Splice isoform 1 of P43686 26S protease regulatory subunit 6B (Proteasome 26S subunit ATPase 4 isoform 1)	PSMC4
128	**IPI00021983**	**0.07**	**Splice isoform 1 of Q92542 Nicastrin precursor**	NCSTN
129	IPI00290416	0.39	Splice isoform 1 of Q9NTK5 Putative GTP-binding protein PTD004 (Obg-like ATPase 1, GTP-binding protein 9)	OLA1
130	**IPI00218319**	**2.15**	**Splice isoform 2 of P06753 Tropomyosin alpha 3 chain (tropomyosin 3 isoform 2)**	TPM3
131	IPI00216704	2.07	Splice isoform 2 of P11277 Spectrin beta chain, erythrocyte*	SPTB
132	**IPI00016786**	**3.82**	**Splice isoform 2 of P21181 Cell division control protein 42 homolog (cell division cycle 42 isoform 1)**	CDC42
133	IPI00220158	0.43	Splice isoform 3 of P35611 Alpha adducin (Erythrocyte adducin subunit alpha)*	ADD1
134	IPI00306667	0.32	Splice isoform CNPII of P09543 2',3'-cyclic nucleotide 3'-phosphodiesterase (CNPase)	CNP
135	IPI00013933	1.81	Splice isoform DPI of P15924 Desmoplakin (desmoplakin isoform I)	DSP
136	**IPI00026299**	**4.03**	**Splice isoform Glycophorin C of P04921 Glycophorin C**	GYPC
137	IPI00292290	0.3	Splice isoform Long of Q08495 Dematin (Erythrocyte membrane protein band 4.9)*	EPB49
138	IPI00029485	0.15	Splice isoform p150 of Q14203 Dynactin 1	DCTN1
139	**IPI00012490**	**1.8**	**Splice isoform XD of P23634 Plasma membrane calcium-transporting ATPase 4 (PMCA4), (Plasma membrane calcium ATPase isoform 4), (Plasma membrane calcium pump isoform 4), (Matrix-remodeling-associated protein 1)**	ATP2B4
140	**IPI00007676**	**1.96**	**Steroid dehydrogenase homolog**	HSD17B12
141	IPI00290566	0.3	T-complex protein 1, alpha subunit (T-complex protein 1 isoform a)*	TCP1
142	IPI00297779	0.17	T-complex protein 1, beta subunit (chaperonin containing TCP1, subunit 2)*	CCT2
143	**IPI00302927**	**1.59**	**T-complex protein 1, delta subunit (chaperonin containing TCP1, subunit 4 (delta)**	CCT4
144	IPI00010720	0.27	T-complex protein 1, epsilon subunit (chaperonin containing TCP1, subunit 5 (epsilon))*	CCT5
145	IPI00018465	0.01	T-complex protein 1, eta subunit (chaperonin containing TCP1, subunit 7 isoform a)*	CCT7
146	IPI00027626	14.38	T-complex protein 1, zeta subunit	CCT6A
147	IPI00023591	0.12	Transcriptional activator protein PUR-alpha (purine-rich element binding protein A)	PURA
148	IPI00000010	21.81	Transforming protein p21b (c-K-ras2 protein isoform b, Kirsten rat sarcoma viral oncogene homolog)	KRAS
149	IPI00027500	10.16	Transforming protein RhoA	RHOA
150	**IPI00022774**	**44.53**	**Transitional endoplasmic reticulum ATPase (TER ATPase) (15S Mg(2+)-ATPase p97 subunit) (Valosin containing protein) (VCP) [Contains: Valosin]**	VCP
151	IPI00328807	0.06	Triosephosphate isomerase*	TPI1
152	IPI00298237	0.01	Tripeptidyl-peptidase I precursor	TPP1
153	**IPI00002375**	**2.88**	**Tropomodulin 1**	TMOD1
154	IPI00387144	0.66	Tubulin alpha-1 chain	TUBA1B
155	IPI00142634	59.23	Tubulin beta-5 chain	TUBB
156	IPI00007074	0.14	Tyrosyl-tRNA synthetase	YARS
157	IPI00026119	0.19	Ubiquitin-activating enzyme E1*	UBA1, UBA1B,
158	**IPI00298337**	**0.38**	**Urea transporter, erythrocyte (solute carrier family 14 (urea transporter), member 2)**	SLC14A2

159	IPI00008219	0.22	UV excision repair protein RAD23 homolog A (RAD23 homolog A)	RAD23A
160	IPI00216311	5.11	Villin 2 (cytovillin 2;, Ezrin) (has high degree of similarity within its N-terminal domain to the erythrocyte cytoskeletal protein, band 4.1, .Goulc et al., EMBO Journal vol.8 no.13 pp.4133-4142, 1989)*	EZR

NB. Proteins shown in bold letters have been reported by Pasini et. al.[8] to be present in RBC membrane and those with asterisks have been reported by Goodman et. al. [7] to be present in whole RBC proteome.
